# Sparse network-based models for patient classification using fMRI

**DOI:** 10.1016/j.neuroimage.2014.11.021

**Published:** 2015-01-15

**Authors:** Maria J. Rosa, Liana Portugal, Tim Hahn, Andreas J. Fallgatter, Marta I. Garrido, John Shawe-Taylor, Janaina Mourao-Miranda

**Affiliations:** a Department of Computer Science, Centre for Computational Statistics and Machine Learning, University College London, London, UK; b Centre for Neuroimaging Sciences, Department of Neuroimaging, Institute of Psychiatry, King's College London, London, UK; c LABNEC, Universidade Federal Fluminense, Rio de Janeiro, Brazil; d Department of Cognitive Psychology II, Johann Wolfgang Goethe University Frankfurt am Main, Germany; e University of Tuebingen, Department of Psychiatry and Psychotherapy, Tuebingen, Germany; f Queensland Brain Institute, The University of Queensland, Brisbane, Australia; g Centre for Advanced Imaging, The University of Queensland, Brisbane, Australia; h Australian Research Council Centre of Excellence for Integrative Brain Function, Australia

**Keywords:** Classification, Sparse models, Gaussian graphical models, Graphical LASSO, L1-norm SVM, Reproducibility/stability, Functional connectivity, Major depressive disorder, fMRI

## Abstract

Pattern recognition applied to whole-brain neuroimaging data, such as functional Magnetic Resonance Imaging (fMRI), has proved successful at discriminating psychiatric patients from healthy participants. However, predictive patterns obtained from whole-brain voxel-based features are difficult to interpret in terms of the underlying neurobiology. Many psychiatric disorders, such as depression and schizophrenia, are thought to be brain connectivity disorders. Therefore, pattern recognition based on network models might provide deeper insights and potentially more powerful predictions than whole-brain voxel-based approaches. Here, we build a novel sparse network-based discriminative modeling framework, based on Gaussian graphical models and L1-norm regularized linear Support Vector Machines (SVM). In addition, the proposed framework is optimized in terms of both predictive power and reproducibility/stability of the patterns. Our approach aims to provide better pattern interpretation than voxel-based whole-brain approaches by yielding stable brain connectivity patterns that underlie discriminative changes in brain function between the groups. We illustrate our technique by classifying patients with major depressive disorder (MDD) and healthy participants, in two (event- and block-related) fMRI datasets acquired while participants performed a gender discrimination and emotional task, respectively, during the visualization of emotional valent faces.

## Highlights

• Connectivity-based predictive models can potentially improve psychiatric diagnosis.
• We present a novel sparse network-based discriminative model for fMRI data.
• This model jointly optimizes predictive power and stability of the output patterns.
• Aiming at better pattern interpretation than voxel-based/whole-brain models.
• Model is applied to classify patients with major depression from two fMRI datasets.

## Introduction

Recent research using pattern recognition methods applied to whole-brain neuroimaging data, such as structural/functional Magnetic Resonance Imaging (s/fMRI) data, has proved successful at diagnosing individual psychiatric patients based on their brain activity and structure (Klöppel et al., 2012; Orrù et al., 2012; Phillips, 2012; Kipli et al., 2013).

In particular, functional studies of major depressive disorder (MDD) have shown high predictive power of pattern recognition models applied to whole-brain task-based fMRI data. For instance, Fu et al. (2008), applied Support Vector Machines (SVM, Cortes and Vapnik, 1995) to discriminate MDD patients from healthy controls, based on patterns of brain activity induced by processing of facial expressions with different levels of sadness. Fu et al. (2008) correctly classified up to 72% of patients and 82% of controls, using all facial stimuli, and up to 84% of patients and 89% of controls, using only neutral faces. Marquand et al. (2008) used a verbal (N-Back) working memory task and SVM to significantly classify 65% of MDD patients and 70% of controls. In addition, within the patients group, the authors classified with 69% accuracy those who responded to treatment and those who did not respond. Similarly, Costafreda et al. (2009a) accurately identified 71% of MDD patients, before treatment, that responded fully to cognitive behavioral therapy (CBT) from whole-brain patterns of brain activity induced once more by a sad facial processing task. Brain structure, including gray and white matter measures, has also been found to be highly predictive of MDD (Costafreda et al., 2009b; Gong et al., 2011; Mwangi et al., 2012; Qiu et al., 2013).

Despite these promising results, whole-brain (voxel-based) predictive models can be difficult to interpret. The first issue relates to the fact that, although whole-brain pattern recognition studies commonly report coordinates for the most discriminative brain regions (Fu et al., 2008; Marquand et al., 2008; Costafreda et al., 2009a), the pattern is not sparse and all voxels in the brain contributed to the predictions. More generally, although it is possible to create voxel-wise maps from the parameters of pattern recognition models, local spatial inferences on these maps are not straightforward. In contrast to univariate models, multivariate maps do not naturally provide a null-hypothesis (and corresponding statistical test) associated with each voxel (Gaonkar and Davatzikos, 2013). Ways to alleviate this issue include feature selection approaches (Guyon and Elisseeff, 2003; Mwangi et al., 2013), or the use of sparse methods, which automatically select, within the pattern recognition model, the most relevant subset of voxels to the predictions (Rasmussen et al., 2012; Grosenick et al., 2013).

The second issue relates to the difficulty in interpreting whole-brain (voxel-based) results in terms of the underlying neurobiology of psychiatric disorders. As is well accepted, many psychiatric disorders, such as MDD and schizophrenia, are thought to be brain connectivity disorders (Konrad and Eickhoff, 2010; Lynall et al., 2010; Hulvershorn et al., 2011; Müller et al., 2011; Zhang et al., 2011; Whitfield-Gabrieli and Ford, 2012; Hulshoff Pol and Bullmore, 2013). In other words, what differentiates these disorders from normal brain function are abnormal connections between brain regions rather that the malfunctioning of a single or set of brain regions alone. This evidence motivates the search for connectivity-based imaging biomarkers of psychiatric disorders. Moreover, in this context, pattern recognition approaches based on brain connectivity models might provide deeper insights and potentially more powerful predictions than whole-brain voxel-based approaches.

Brain connectivity analyses for functional MRI data can be divided into two groups (Friston, 1994): functional connectivity measures, which assess statistical dependencies between signals from distributed brain regions (Van Den Heuvel and Hulshoff Pol, 2010; Varoquaux and Craddock, 2013), and effective connectivity measures, which assess networks of causal effects of one region over another (Friston et al., 2003; Deshpande et al., 2009). Here we focus on functional connectivity approaches for fMRI (fcMRI). In the context of pattern recognition-based predictive models, fcMRI-derived features have recently been successfully used to identify network-based biomarkers of schizophrenia (Cecchi et al., 2009; Shen et al., 2010), Alzheimer's disease and mild cognitive impairment (Stonnington et al., 2010; Wee et al., 2012), autism (Anderson et al., 2011; Nielsen et al., 2013), attention-deficit–hyperactivity-disorder (ADHD, Zhu et al., 2008) and brain maturation (Dosenbach et al., 2010). In the context of depression, Craddock et al. (2009) used SVM and resting state fMRI to compare different feature selection approaches for classifying MDD patients based on the pair-wise correlation between the signals of 15 regions of interest. More recently, Zeng et al. (2012) and Cao et al. (2014) used SVM in combination with univariate feature selection procedures on resting-state fMRI data, to successfully classify MDD patients and identify the most discriminative networks from all possible pair-wise correlations between anatomically defined regions.

These results have shown that pattern recognition techniques are well suited for measuring whether discriminative information about psychiatric disorders, and MDD in particular, exists in distributed brain networks. However, the majority of modeling approaches used to date do not directly (within the feature extraction and predictive model) identify the connections that are most relevant to the predictions, without relying on ad-hoc and often time-consuming feature selection approaches (Craddock et al., 2009; Zeng et al., 2012).

On the feature extraction side, one way of minimizing the number of connections between brain regions is to estimate the sparse inter-regional inverse covariance matrix (Friedman et al., 2008). Sparsity is imposed via an L1-norm penalty on the connection estimates and the zero entries in this matrix correspond to conditional independence between the signals of two brain regions, given all others. These matrices also define Gaussian graphical models, where a missing edge between two nodes is equivalent to a zero entry in the inverse covariance matrix. This method can be useful to select only a subset of relevant connections and has been shown to be more sensitive to detect underlying networks, under different signal conditions, than other functional connectivity approaches, such as full correlation-based approaches (Smith et al., 2011).

Sparse inverse covariance-based features have been used in classification problems of mental illnesses (Cecchi et al., 2009; Bohland et al., 2011; Wee et al., 2012) but, to our knowledge, they have not yet been combined with a sparse discriminative classifier to provide a fully (from feature extraction to prediction) sparse modeling framework for the classification of patients suffering from psychiatric disorders. In this paper, we extend previous efforts on combining the two (Rosa et al., 2013). We build a novel connectivity-based discriminative framework combining sparse inverse covariance-based features (Friedman et al., 2008) estimated from task-based fMRI data and L1-norm regularized linear Support Vector Machines (SVMs, Fan et al., 2008) for classification. The advantage of combining these two approaches is two-fold: linear L1-norm SVMs are very efficient on large sparse datasets, as opposed to more commonly used L2-norm SVMs (Mourão-Miranda et al., 2005; Orrù et al., 2012) and yield a sparse linear decision boundary, revealing only a small set of features that best discriminate the two groups (Fan et al., 2008).

Previous work has been published where sparse connectivity-based features and classification is jointly optimized. Zhou et al. (2014) developed an optimization framework to maximize the discriminative power of graphical LASSO-based generative models. The authors applied this framework to Alzheimer's patient classification using Positron Emission Tomography (PET) data. Eavani et al. (2014) jointly optimized a factorization of correlation matrices into small networks with an L2-norm SVM to discriminate between healthy children and adults using resting state fMRI. Although these frameworks also provide sparse connectivity-based discriminative patterns, the stability/reproducibility of the solution was not considered. Since the primary goal of this work is better pattern interpretation, it is important to take into account the reproducibility of the model parameters (pattern), in terms of how much these parameters overlap when estimated with different subsamples of the data. For this purpose, Rasmussen et al. (2012) proposed a model evaluation scheme where both the predictive power and reproducibility of the model are jointly optimized, using a split-half subsampling approach of the data. Here we use a similar procedure to optimize the L1-norm SVM. We use not only its accuracy but also the reproducibility, defined as the overlap between sparse patterns across cross-validation folds (also referred to as stability), of its solution. To the best of our knowledge, stability measures, such as the mean overlap proposed in Baldassarre et al. (2012) and/or stability selection (Meinshausen and Bühlmann, 2010; Ryali et al., 2012) have not yet been used in connectivity-based predictive models of fMRI data.

The framework proposed here therefore imposes two sparsity levels: one at the features and one at the classification (model parameters) level. By imposing these two sparsity levels we posit that task-induced neuronal processing involves only a discrete number of connections between brain regions, from which only a subset is affected by the condition being discriminated (e.g. depression).

We apply our technique to two fMRI datasets acquired from two different samples of patients with symptoms of MDD and matched healthy participants. The first dataset has an event-related design involving implicit processing of sad faces of different emotional intensity. This dataset was used in Rosa et al. (2013) to test a prior version of our framework. The second dataset comprises a block-related design experiment in which participants viewed faces of different emotional content, including happy, anxious, neutral and sad faces. We show that the resulting pattern from our sparse network-based classification framework has a more straightforward interpretation than whole-brain (voxel-based) patterns as it finds a biologically meaningful multivariate network signature that best differentiates MDD patients from controls.

In addition, we compare our approach to commonly used correlation and partial correlation based metrics for functional connectivity.

This paper is organized as follows. The next section describes the fMRI data and the sparse network-based pattern recognition framework. We then present classification results for different network-based features and the set of most discriminative connections for the sparse inverse-covariance based models. Finally, we discuss the limitations of our approach and interpret our results in light of previous work from the literature.

## Materials

### Event-related fMRI dataset

#### Participants and task

We use the same fMRI dataset of Fu et al. (2004, 2008), from nineteen medication-free patients (13 women; mean age 43.2 years; standard deviation (SD) 8.8 years) and nineteen controls (11 women; mean age 42.8 years; SD 6.7 years), matched by age and intelligence quotient (IQ). Patients were diagnosed with major depressive disorder (score of at least 18 on Hamilton Rating Scale for Depression) according to clinical interviews with a psychiatrist. The project was approved by the Ethics Research Committee, Institute of Psychiatry, London, England.

The experimental task followed an event-related design involving images of faces with three different levels of emotional intensity (low, medium, and high intensity of sadness) and baseline trials (crosshair fixation), which were presented in random order for 3 s each (mean inter-trial interval of 5 s). Each facial stimulus was presented twice at the same intensity (60 faces total), along with 12 baseline trials, for a total of 72 trials. For each face trial, participants were asked to indicate the gender of the face with a joystick. This task design was used to elicit incidental (not explicit) affective processing. More information on the patients' demographic features and experimental task can be found in the original studies (Fu et al., 2004, 2008).

#### fMRI acquisition and analysis

Gradient-echo single-shot echo-planar imaging was used to acquire 180 T2-weighted image volumes for each participant on a neuro-optimized 1.5 T IGE LX System (General Electric, Milwaukee, Wisconsin) at the Maudsley Hospital, South London and Maudsley National Health Services (NHS) Trust, London. For each volume, 16 noncontiguous axial planes parallel to the intercommissural plane were collected with the following parameters: repetition time 2 s; echo time 40 ms; section thickness 7 mm; skip .7 mm; in-plane resolution 3 × 3 mm. 180 scans were used for the analyses.

The data were realigned, normalized to the Montreal Neuroimaging Institute (MNI) template and smoothed (using an 8 mm Gaussian kernel) using SPM2 (Wellcome Trust Centre for Neuroimaging, UK) as described in Fu et al. (2004, 2008).

### Block-related fMRI dataset

#### Participants and task

This dataset was collected for a previous study by Hahn et al. (2011). Thirty patients (18 males, mean age 38.1 years, SD 11.0 years) from the Department of Psychiatry, Psychosomatics, and Psychotherapy at the University of Wuerzburg, Germany, diagnosed with recurrent depressive disorder, depressive episodes, or bipolar affective disorder on the basis of the consensus of two psychiatrists participated in the study. Patients were recruited on a variety of medications and, at the time of the measurement procedures, presented varying degrees of depressive symptoms (from severe to almost symptom free). Having a well-diagnosed but heterogeneous group of patients with varying degrees and types of medication provides a way of accounting for the medication confound (Hahn et al., 2011). Thirty control participants (19 males; mean age 36.0 years; SD 9.1 years) recruited from the local population were selected to match the patient group for sex, age, smoking status, and handedness. Written informed consent was obtained from all 60 participants and the study was approved by the ethics committee of the University of Wuerzburg.

The experimental task followed a block-related design consisting of passively viewing emotional faces. Sad, happy, anxious, and neutral facial expressions were used. Each block contained pictures of faces from 8 individuals (four female). Each face was shown against a black background for 2 s and was immediately followed by the next face. The pictures were obtained from the Karolinska Directed Emotional Faces database. Every block was randomly repeated 4 times and lasted 16 s. Face blocks were alternated with blocks of the same length showing a white fixation cross on which the participant had to focus. Participants were instructed to attend to the faces and empathize with the emotional expression. In contrast to the previous task, this design was used to elicit explicit (not incidental) affective processing. A more detailed description of the patients and experimental task can be found in the original study (Hahn et al., 2011).

#### fMRI acquisition and analysis

Imaging was performed using a 1.5-T Magnetom Avanto total imaging matrix MRI scanner (Siemens, Erlangen, Germany) equipped with a standard 12-channel head coil. In a single session, twenty-four 4-mm-thick, interleaved axial slices (in-plane resolution, 3.28 × 3.28 mm) oriented at the anterior commissure - posterior commissure transverse plane were acquired with a 1 mm inter-slice gap, using a T2*-sensitive single-shot echo planar imaging sequence with the following parameters: repetition time, 2 s; echo time, 40 ms; flip angle, 90°; matrix, 64 × 64; and field of view, 210 × 210 mm^2^. The first 6 volumes were discarded to account for magnetization saturation effects. The following 256 scans were used for the analyses. Stimuli were presented via MRI-compatible goggles (VisuaStim; Magnetic Resonance Technologies, Northridge, California).

The data were realigned, normalized to the MNI template and smoothed (using an 8 mm Gaussian kernel) using SPM5 (Wellcome Trust Centre for Neuroimaging, UK) as described in Hahn et al. (2011).

### Regional mean time-series

**Fig. 1:**
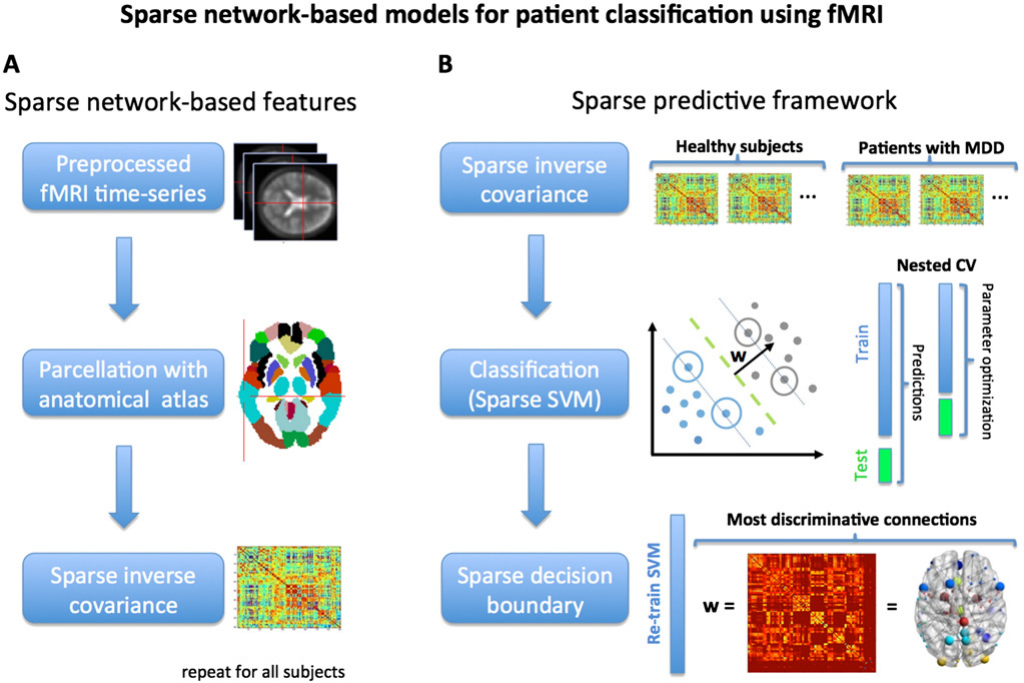
Sparse network-based predictive models for patient classification. Panel A: sparse network-based features. The preprocessed fMRI time-series are parcellated into regions using an anatomical atlas. From the regional time-series we then compute pair-wise covariance matrices. From these matrices we estimate the sparse inverse covariance using graphical LASSO. We use these as features for classification (see Panel B). This procedure is done separately for each participant. Panel B: sparse predictive model. We then feed the sparse inverse covariance matrices into a sparse SVM framework for classification. We use nested cross-validation to make predictions and optimize parameters (i.e. the inner loop was used for parameter optimization and the outer loop was used to make the predictions). Optimization is therefore performed using only training data. The resulting decision boundary is sparse and yields the set of most discriminative brain connections between patients and controls.

In order to extract functional connectivity-based features for classification, the fMRI volumes from both datasets (*n_t_* time-points × *n_d_* voxels) were parcellated into *n_p_* regions using an anatomical atlas (Fig. 1). Regional mean time-series (*n_t_* time-points × *n_p_* regions) were estimated by averaging the fMRI signals over all voxels within each atlas region. Here we used the sulci probabilistic atlas from BrainVISA^1^ (Perrot et al., 2009) to define 122 cortical regions and the Harvard-Oxford atlas^2^ for 15 subcortical regions. The total number of regions, *n_p_*, is therefore 137. We chose to use the sulci probabilistic atlas instead of a more traditional atlas, such as the Automated Anatomical Labeling (AAL) atlas, because as opposed to the AAL atlas, the sulci atlas is multi-subject and probabilistic-based, and has been shown to provide good support to define regions of interest in fMRI studies (Keller et al., 2009).

To make sure that we fully removed the effects of movement, the parameters from the realignment step of the preprocessing (*n_t_* time points × 6 (3 rotation + 3 translation) parameters) were regressed out of the averaged regional time-series using a residual forming matrix (Worsley and Friston, 1995). We note here that in functional connectivity analyses, in particular for resting state data, there is still no consensus on whether to regress out other confounds, such as the global signal (Fox et al., 2009;Rubinov and Sporns, 2010). This issue is however more critical for resting state (as opposed to task-based) fMRI since many physiological sources of noise overlap mostly with low-frequency BOLD fluctuations that characterize resting state networks (Murphy et al., 2009). In this work, we used only task-based fMRI data, and to increase sensitivity, we chose not to correct for the global signal and other confounds. In addition, we did not regress out the task stimuli from the regional signals, since our goal was to detect changes in brain connectivity induced by the emotional task that allow us to discriminate the two groups.

After this step, the averaged motion-corrected time-series were filtered for low frequency components, using a set of five Discrete Cosine Transforms with a cut off period of 128 s. The whole procedure was done independently for each participant.

After parcellation, motion correction and filtering we computed different functional connectivity measures from the regional time-series.

## Methods

In this section, we present a novel connectivity-based sparse framework for classification using fMRI data. We first describe how to extract functional connectivity-based features from fMRI, using sparse inverse covariance models and other correlation-based metrics. We then present the linear classifiers used for prediction.

### Feature construction

#### Sparse network-based features

From the regional time-series (*n_t_* time-points × *n_p_* regions), we can compute the (*n_p_* regions × *n_p_* regions) pairwise inter-regional covariance matrix, Σ, for each participant. From this covariance matrix we can then estimate sparse functional brain networks using Gaussian graphical models.

Graph theory has proved very useful to describe statistical dependencies between random variables (Koller and Friedman, 2009). A graph is a mathematical object defined by a pair *G* = (*V*, *E*), in which *V* is a set of nodes (e.g. brain regions), and *E* is a set of edges connecting pairs of nodes (e.g. functional connectivity between brain regions). Gaussian graphical models, in particular, assume that the variables have a multivariate Gaussian distribution with mean *μ* and covariance Σ. In addition, if the edge linking nodes *j* and *i* is absent, then nodes *j* and *i* are conditionally independent given all the others, and the corresponding entry of the inverse covariance matrix, Ω = Σ^− 1^, is zero.

One can therefore estimate functional connectivity between brain regions using Gaussian graphical models by estimating the sparsity pattern of the inverse covariance matrix, Ω. Here we used the graphical Least Absolute Shrinkage and Selection Operator (LASSO)^3^ approach for estimating these graphs (Friedman et al., 2008). Graphical LASSO tries to find a positive definite matrix, Ω, which maximizes the penalized Gaussian log-likelihood:

(1) $$L\left(\Omega \right)-\lambda {\left\|\Omega \right\|}_{1}=\log\det\Omega -tr\left(\Omega \Sigma \right)-\lambda {\left\|\Omega \right\|}_{1}\text{,}$$

from the sample covariance matrix, Σ. *Log det* and *tr* correspond to the logarithm of the determinant, and the trace of the matrix, respectively. ||.||_1_ is the matrix L1-norm (sum of absolute values of all entries in the matrix) and *λ* is a regularization parameter, which controls the amount of sparsity (zero elements) in the estimate of Ω. Graphical LASSO uses the block-coordinate descent optimization algorithm proposed by Friedman et al. (2008). In each descent step, the algorithm estimates a single row (and column) of Ω by solving a modified LASSO regression problem: the *i*, *j* element of Σ^− 1^ is, up to a constant, the regression coefficient of node *j* in a multiple linear regression of node *i* on all other nodes (Tibshirani, 1996). We emphasize here that sparsity is determined only by the regularization term in graphical LASSO and not by additional thresholding.

**Fig. 2:**
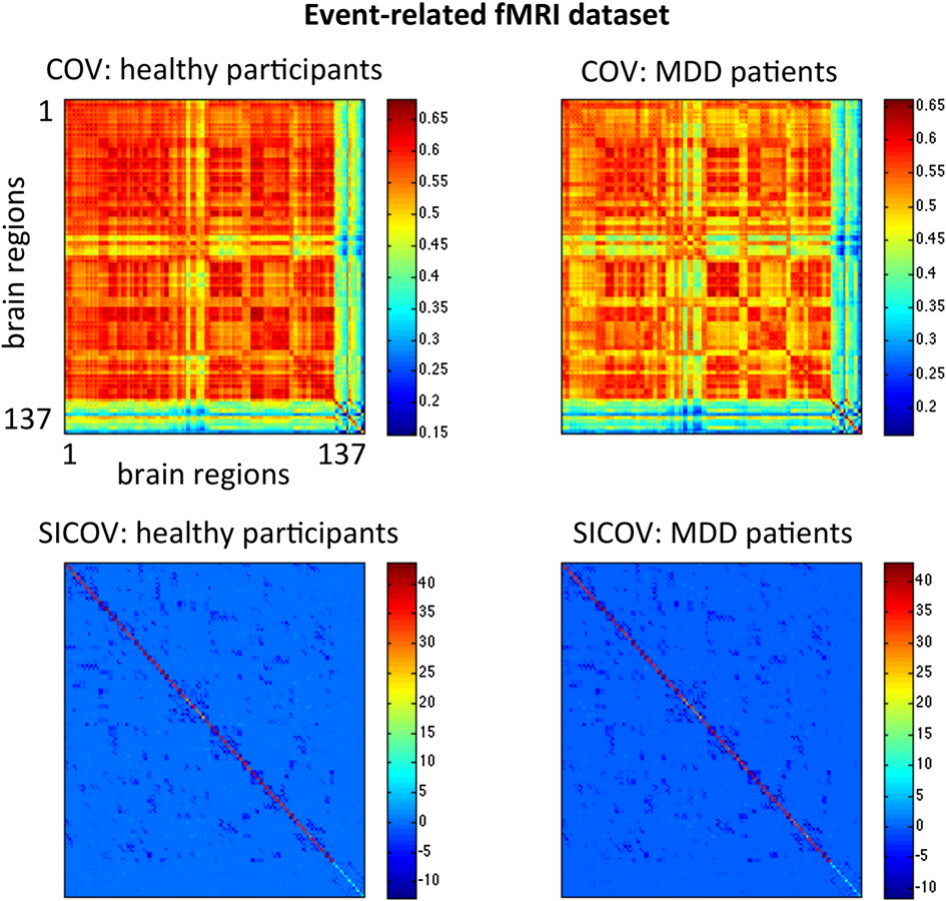
Covariance (COV) and sparse inverse covariance (SICOV, *λ* = 0.01) matrices from the healthy participants and patients with MDD for the event-related fMRI dataset. The covariance and inverse covariance matrices were computed by pooling the time-series of all participants together for illustration purposes only.

**Fig. 3:**
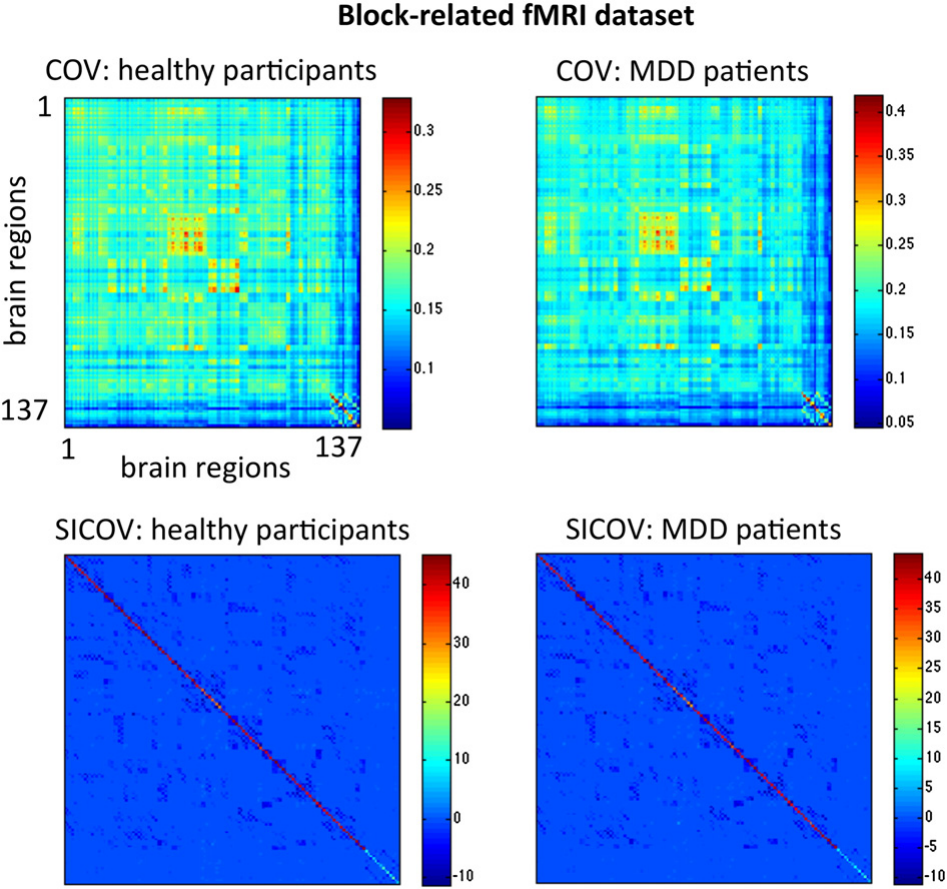
Covariance (COV) and sparse inverse covariance (SICOV, *λ* = 0.01) matrices from the healthy participants and patients with MDD for the block-related fMRI dataset. The covariance and inverse covariance matrices were computed by pooling the time-series of all participants together for illustration purposes only.

A graph is equivalent to its adjacency matrix, which in this case is given by the sparse inverse covariance matrix, Ω. The graphs estimated here are undirected (Ω is symmetric) and weighted (Ω is a real-valued matrix, as opposed to binary). Examples of these matrices are shown in Figs. 2 and 3.

#### Non-sparse network-based features

We used other common functional connectivity measures for comparison with the sparse inverse covariance matrices described in the previous section. The simplest non-sparse measure is the pair-wise Pearson's correlation coefficient, between brain regions, Φ. We also used the full (non-sparse and non-regularized) inverse covariance, $\Pi \overset{def}{=}\Sigma^{-1}$, for comparison. We note here that the covariance matrix can be directly inverted when the number of nodes (brain regions) is smaller than the number of time points, which is true for our data. When this is not the case, the covariance matrix is singular and therefore not directly invertible. The third measure is partial correlation, Θ, which is the normalized correlation between two time-series, after each has been adjusted by regressing out all other time-series in the data. This measure is related to the inverse covariance matrix as follows: each entry *i*, *j* of Θ is equal to $\frac{-\Pi_{ij}}{\sqrt{\Pi_{ii}\Pi_{jj}}},\;i\neq j$.

### Pattern classification

#### Linear L1-norm regularized SVM

Given the functional networks obtained in the previous step, we then use a sparse supervised learning framework for participant classification (Fig. 1). Supervised learning approaches for binary classification try to find a relationship between training inputs, *x*_*i*_ ∈ ℜ^*q*^, and their corresponding label, y_*i*_ = {− 1, + 1} (e.g. control and patient), by estimating a prediction function *f*(*x*_*i*_) : ℜ^*q*^ → ℜ, where *i* is a training sample and *q* is the dimensionality of *x*. For linear algorithms this function, also known as decision function, can be written as *f*(*x*_*i*_) = *sign*(*w*^*T*^*x*_*i*_), where *w* ∈ ℜ^*q*^ is a vector of coefficients, known as the weight vector, to be estimated. If *sign*(*w*^*T*^*x*_*i*_) > 0 the input *x*_*i*_ is classified as belonging to class 1 (e.g. patient) and if *sign*(*w*^*T*^*x*_*i*_) < 0 it is attributed to class 2 (e.g. healthy participant). In sparse models, some of the entries of *w* are set to zero (e.g. through regularization), which can potentially aid interpretation since only a subset of the input features are selected as being relevant for the predictive model. Here we used a sparse classification approach based on linear L1-norm regularized Support Vector Machines (SVM). In this section, we briefly introduce these machines, as well as the cross-validation and performance criteria used to evaluate the model.

Linear L1-norm regularized SVM (Fan et al., 2008) are a binary maximum margin classifier (Fig. 1), which yields a sparse weight vector, *w* by solving the following optimisation problem:

(2) $$\min_{w}f\left(w\right)\overset{def}{=}{\left|\left|w\right|\right|}_{1}+C\sum_{i=1}^{n_{k}}\xi \left(w;x_{i},y_{i}\right)\text{.}$$

The parameter *C* > 0 controls the trade-off between the width of the margin separating the two classes and the number of misclassified examples, and *n*_*k*_ is the number of training examples. L1-norm SVM is a non-kernel method. Therefore it works in the original feature or input space and not in a feature space defined by a kernel function, as the most commonly used L2-norm SVM (Cortes and Vapnik, 1995; Chang and Lin, 2011). In our connectivity-based framework, the features that comprise the training examples, *x*_*i*_ ∈ ℜ^*q*^, represent the vectorized lower triangular entries of the functional connectivity matrices (Ω, Φ, Π, and Θ) described above, where *q* = *n*_*p*_(*n*_*p*_ − 1)/2. We note here that for the sparse matrices, L1-SVM was trained on the entire lower-triangular matrix (including both zero and non-zero entries) and not on the union of the non-zero entries in the matrices across different subjects. L1-SVM is particularly well suited for high-dimensional sparse data (Fan et al., 2008). The class labels *y*_*i*_ are either + 1 for patients with major depression symptoms or − 1 for healthy participants. To train SVM, we used the following squared loss function in Eq. (2):

(3) $$\xi \left(w;x_{i},y_{i}\right)=\max{\left(1-y_{i}w^{T}x_{i},0\right)}^{2}$$

We used the LIBLINEAR^4^ software package to solve the SVM optimization problem (Fan et al., 2008).

#### Linear L2-norm SVM

In order to further test our initial hypothesis that only a small set of connections best discriminates the patients from the controls, we compare the sparse L1-norm SVM to the more commonly used non-sparse L2-norm SVM (Chang and Lin, 2011). L2-norm SVM is a maximum margin binary classifier that yields a non-sparse weight vector, *w*. It has been extensively used in neuroimaging (Mourão-Miranda et al., 2005; LaConte et al., 2005; Magnin et al., 2009; Meier et al., 2012; Gould et al., 2014) and its formalism has been described in detail in Mourão-Miranda et al. (2005), Lemm et al. (2011) and other works. For a critical review see Orrù et al. (2012). Here we used a linear kernel L2-norm SVM as implemented in the LIBSVM^5^ software toolbox and exactly the same nested-cross validation scheme used for L1-norm SVM, as described below.

### Nested cross-validation

#### Cross-validation framework

To train the model, we used the following nested cross-validation (CV) scheme (Fig. 1). We implemented an outer leave-one-subject-per-group-out (LOSGO) cross-validation framework to make predictions (i.e. classify patients and controls) using fixed parameters *C* (for the SVM) and λ (when using the graphical LASSO). This means that in every fold we leave two test participants out (one from each group) and train the model (i.e. estimate the model parameters) with the remaining participants. The total number of outer folds is therefore the number of participants in each group, *n_s_*. As mentioned above, the input features for each participant are the vectorized lower triangular entries of the connectivity matrices.

Inside each of the outer CV folds, we run another LOSGO-CV loop to optimize the *C* and λ (when using graphical LASSO) parameters. The inner CV loop contains a total of *n_s_* − *1* folds, where *n_s_* is the number of participants in each group. The inner CV loop does not contain the two participants left out in the outer CV loop. This guarantees a complete separation of training and testing data for both optimization and prediction.

#### Graphical LASSO parameter optimization

To optimize the graphical LASSO parameter *λ* we use the Bayesian Information Criterion (BIC) (Schwarz, 1978):

(4) $$BIC\left(\lambda \right)=-2L\left(\Omega \left(\lambda \right)\right)+d\left(\lambda \right)\log n_{t}\text{,}$$

where L(Ω(*λ*)) is the log-likelihood function as defined in Eq. (1) and d(*λ*) are the degrees of freedom. A common practice is to calculate d(*λ*) as d(*λ*) = *m*(*λ*)(*m*(*λ*) − 1)/2, where *m*(*λ*) is the number of non-zero elements of Ω for a given value of *λ*. We estimate one Ω matrix for the patients and one for the controls separately by concatenating (in time) the data from all participants in each group. We then find *λ* (from 0.1, 0.01 and 0.001) that more frequently minimizes Eq. (4) in the inner CV folds for each group. We then use the average of the parameters chosen for each group to estimate Ω for each participant individually in the outer CV loop. We chose three values for *λ* mainly for computational reasons but since they span a wide range of sparsity levels (from almost full to very sparse) we believe that these values are sufficient for our subsequent analyses.

#### L1-norm SVM *C* parameter optimization

The *C* parameter is optimized by varying its value between 10^− 5^ to 10^5^ (in logarithmic steps) and then finding *C* that more frequently maximizes simultaneously the accuracy and reproducibility/stability of the pattern in the inner CV folds.

Here we use the definition of stability introduced in Baldassarre et al. (2012). Let *β*(*s*) be the weight vector estimated in one of the inner CV folds when the set of subjects *s* are left out. The model support can then be defined as *I*_*s*_ := {*i*|*β*(*s*)_*i*_ ≠ 0} as the index set of the location of non-zero weights. The model sparsity can be defined as the relative number of non-zero weights, $S\left(s\right):=\frac{\left|I_{s}\right|}{q}$, and the corrected pairwise relative overlap between the weights of two different folds as:

(5) $$O_{s,s'}:=\frac{\left|I_{s}\cap I_{s^{\prime }}\right|-E}{\max\left(\left|I_{s}\right|,\left|I_{s'}\right|\right)}\text{.}$$

where *q* is the total number of weights (features) and *E* is the expected overlap between the support of two random vectors with sparsity *S*(*s*) and *S*(*s* ′), respectively: *E* = *S*(*S*)*S*(*s* ′)/*q*. *Stability* (*reproducibility*) is then computed as the average overlap across all inner cross-validation folds N:

(6) $$\bar{O}:=\frac{1}{N\left(N-1\right)}\sum_{s\neq s\prime =1}^{N}O_{s,s\prime }\text{.}$$

Finally to select the parameter *C* to be used in the outer CV loop we minimize the distance metric proposed by Strother et al. (2002) and defined as follows:

(7) $$D=\sqrt{{\left(1-Acc\right)}^{2}+{\left(1-\bar{O}\right)}^{2}}$$

where *Acc* is the accuracy of the model and *Ō* is the stability measure defined in Eq. (6). Both these values are calculated inside the inner CV loop.

### Model evaluation

There are different ways of assessing the generalization performance of a classifier. Here, we use the accuracy, which is defined as the number of correctly classified test examples divided by the total number of test examples, averaged over all outer cross-validation folds. We also measure the sensitivity and specificity, which are commonly used in clinical classification problems. These estimates can be obtained as follows:

(8) $$\begin{array}{l} \mathrm{Sensitivity}=\;\frac{TP}{TP+FN}\\ \mathrm{Specificity}=\;\frac{TN}{TN+FP} \end{array}$$

where *TP*, *FP*, *TN* and *FN* represent the number of true positives (patients classified as patients), false positives (controls classified as patients), true negatives (controls classified as controls), and false negatives (patients classified as controls), respectively.

To assess whether the estimated accuracy differs from what is expected if the classifier was randomly assigning labels we used permutation tests. By permuting the labels (i.e. assigning a label *y*_*i*_ = {− 1, + 1} randomly for each example *x*_*i*_) and re-running the classification framework every time we permute the labels, we can estimate the distribution of the accuracy under the null hypothesis (i.e. that we have a random classifier). The probability of obtaining a given or more extreme value of the accuracy under the null hypothesis (p-value) can then be estimated by dividing the number of times, n_*l*_, the accuracy obtained with the permuted labels is equal or higher to the value of the accuracy estimated with the true labels, divided by the total number of permutations, n_*p*_, (i.e. p-value = $\max\left(\frac{1}{n_{p}},\frac{n_{l}}{n_{p}}\right)$).

### Pattern interpretation

The weight vector, *w*, defines the decision boundary of the linear classifier (i.e. the optimal separating hyperplane between the two classes) and its dimensionality equals that of the input feature vectors, *w* ∈ ℜ^*q*^. Therefore, each entry of *w* corresponds to a particular feature, in this case a functional connectivity measure between two brain regions. The magnitude of the elements of the weight vector can thus be interpreted as the contribution of each connection to the separation of the classes. However, it is important to note that the predictions are based on all non-zero features.

As mentioned above, the weight vector from our discriminative modeling framework is sparse and each cross-validation fold yields a slightly different vector (different connections will be zero). To recover the overall set of the most discriminative connections, we retrain the model using the entire dataset and the median value of the parameters optimized within the nested cross-validation. We note here that it is common practice in statistics to estimate a model using the entire dataset once over-fitting has been accounted for using cross-validation or another approach (Hastie et al., 2009).

To obtain the set of connections, which have a high probability of contributing to the predictions, we use the same permutation testing approach used to test the significance of the accuracy. By permuting the labels and re-running the whole classification framework (including the nested cross-validation) we can generate a null distribution of the weights associated with each connection. Comparing the value of the weight obtained using the correct labels with the corresponding null distribution allows one to estimate its statistical significance (i.e. its probability of contributing to the predictions according to the permutation test). Given the amount of tests necessary to make inferences on all connections, we false discovery rate (FDR)-corrected the tests for multiple comparisons (p-value < 0.05).

The resulting statistically significant elements of *w* comprise a distributed connectivity signature that can discriminate patients from controls.

## Results

In this section, we present and compare the performance of the connectivity-based classifiers using the two fMRI datasets described in previous sections. We classify patients with symptoms of major depression and healthy participants and, for the sparse inverse covariance-based L1-norm classifiers,^6^ we present the set of connections that best discriminates the two groups during processing of emotional faces.

### Event-related fMRI dataset

#### Pattern classification

The sparse network models, based on the sparse inverse covariance, which was estimated with graphical LASSO, correctly classified 68% of patients and 89% of controls from the event-related dataset, corresponding to a total accuracy of 79% (p-value = 0.02, permutation test with 100 repetitions, Table 1). In comparison, correlation, inverse covariance and partial correlation-based measures did not perform better than chance. Correlation-based features, correctly classified 84% of patients but only 47% of controls, total accuracy of 66% (p-value = 0.05, permutation test with 100 repetitions). Partial correlation correctly classified 47% of patients and 21% of controls, corresponding to an accuracy of 34% (p-value > 0.05, permutation test with 100 repetitions). Full inverse covariance-based features correctly classified only 32% of patients and 26% of controls, total accuracy of 29% (p-value > 0.05, permutation test with 100 repetitions). The classification results are summarized in Table 1.

The results obtained with the L2-norm SVM are summarized in Table 2. As can be seen, using sparse inverse covariances as features yielded an accuracy of 74% (with 74% sensitivity and specificity) for the event-related dataset (p-value = 0.01, permutation test with 100 repetitions, Table 2). Full correlation-based features yielded an accuracy of 68% (84% sensitivity but only 53% specificity; p-value = 0.02, permutation test with 100 repetitions, Table 2). However, inverse covariance and partial correlation-based measures did not perform better than chance. Partial correlation correctly classified 66% of subjects (p-value > 0.05, permutation test with 100 repetitions) and full inverse covariance-based features correctly classified only 45% of subjects (p-value > 0.05, permutation test with 100 repetitions).

#### Pattern stability/reproducibility

In addition to the predictive accuracy we also present the stability measure (overlap across all inner cross-validation folds, Eq. (6), averaged across all outer CV folds) for all connectivity features and classifiers (Tables 1 and 2). Since the L2-norm SVM is a non-sparse classifier, stability was calculated by setting the smallest non-zero weights, corresponding to 1% of the L1-norm of *w*, to zero, as proposed in Baldassarre et al. (2012). We also added the amount of sparsity (i.e. the mean percentage of non-zero weights across all folds) for the L1-norm SVM (the L2-norm SVM provides a non-sparse weight vector). As can be seen in Tables 1 and 2, L1-norm SVMs provided more stable patterns than L2-norm SVMs (54.02% ± 6.00% compared to 37.81% ± 0.26%, respectively) using the sparse inverse covariance as features. The same seems to be true for all other connectivity measures. One thing to note is that although the stability of the non-sparse correlation-based metrics seems to be higher than the sparse inverse covariance for the L1-norm SVM, conclusions cannot be taken since none of these metrics showed a significant relationship between the data and the labels (predictive accuracies p-value > 0.05 for all measures).

#### Pattern interpretation

As described in the Methods section, the weight vector (that defines the decision boundary of the classifier) yielded by the L1-norm SVM is sparse and the non-zero elements, in this case, can be interpreted as the most discriminative connections between patients with MDD and healthy participants. After re-training the classifier using the sparse inverse covariance-based features and the entire even-related fMRI dataset (SVM parameter *C* = 10, obtained as described in the Methods section), we obtained a set of 62 (out of 9316 possible) connections. The λ parameter (Eq. (1)) did not vary across folds and we therefore used the optimal value of 0.01 to re-train the model. To test the significance of these connections we ran a permutation test on the weights (100 samples) as described in Methods. The stability of the final pattern (output of Eq. (6)) obtained with L1 and L2-norm SVM, measured using a leave-one-subject-per-group-out CV and C = 10 and 0.01, respectively, is plotted in Fig. 6.

**Fig. 4:**
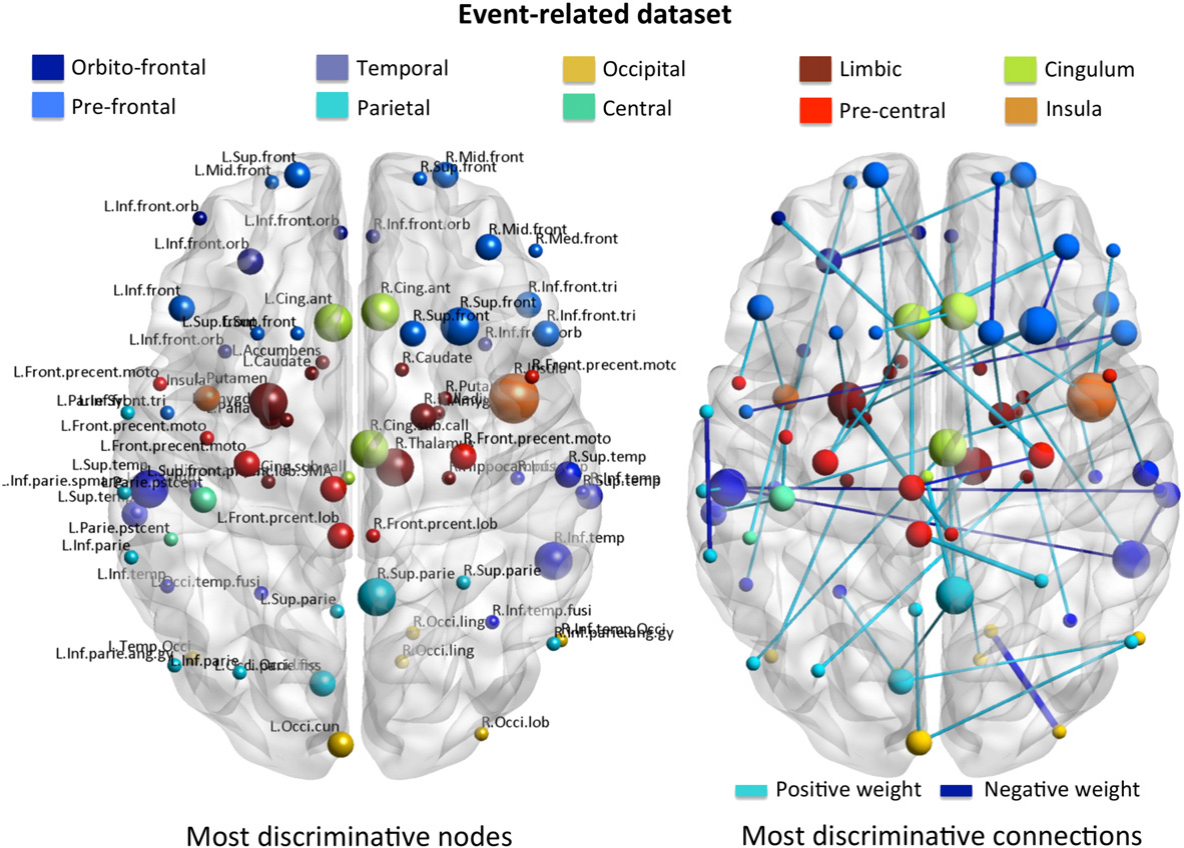
A. Set of most discriminative nodes for the event-related fMRI dataset. The size of the node is proportional to the number of connections that link the corresponding node to others (visualized with BrainNet Viewer). B. Set of most discriminative connections (weight vector) for the event-related dataset. The width of the connection is proportional to the absolute value of the corresponding weight. BrainNet Viewer: http://www.nitrc.org/projects/bnv/.

The resulting network with 59 statistically significant connections (p < 0.05 FDR corrected) is shown in Table 3 and Fig. 4B. The majority of these connections involve *limbic-cortical*, in particular *striatal-cortical*, circuitry and include links between the: left putamen and right pre-central cortex; right pallidum and right inferior frontal cortex; left putamen and right superior frontal cortex; right amygdala and right inferior temporal cortex; left caudate and left superior frontal cortex; right pallidum and superior frontal cortex; right putamen and right inferior temporal cortex; right caudate and left superior frontal cortex; right thalamus and left pre-central cortex; left amygdala and left pre-central (motor) cortex; left putamen and left superior frontal cortex; left amygdala and right superior frontal cotex; left nucleus accumbens and left pre-central (motor) cortex. In addition, the network also highlights *cingulate-cortical connections* (left anterior cingulate cortex and right middle frontal cortex; left anterior cingulate cortex and left supplementary motor area; right anterior cingulate cortex and left superior frontal cortex), *limbic-cingulate connections* (right anterior cingulate cortex and right thalamus; left anterior cingulate cortex and left pallidum), as well as *insular-limbic/cortical/cingulate connections* (right insula and right inferior frontal cortex; left insula and left parietal postcentral cortex; right insula and right medial frontal cortex; left insula and left inferior frontal cortex; right insula and superior temporal cortex; right insula and right anterior cingulate cortex).

From the set of connections obtained, we can determine the set of most discriminate nodes (i.e. regions with more connections). These results are shown in Fig. 4A. The most discriminative node (with 4 connections) for the event-related fMRI dataset is located in the right insula. With a 3-node degree we then obtained the: left and right anterior cingulate cortex, left putamen, right thalamus, left and right subcallosal cortex, right superior frontal cortex, right inferior temporal cortex, left superior temporal cortex and the right superior parietal cortex. The rest of the nodes shown in Fig. 4A connect with two or one node each.

It is important to note here that even though we have highlighted well-known sub-networks and nodes with the highest degree of connectivity from the set of most discriminative connections, all 62 connections and corresponding regions are part of the distributed response that makes the predictions. The full list of connections and corresponding coordinates in the atlas can be found in Table 3.

Patterns for the L2-norm SVM (for both datasets) are not displayed since they are dense (all 9316 connections are present in the final pattern) and therefore extremely difficult to interpret without introducing any post-hoc measures to reduce their complexity. This is however outside of the scope of this paper.

### Block-related fMRI dataset

#### Pattern classification

We obtained similar results using the block-related fMRI dataset. Our sparse network models, based on the sparse inverse covariance, correctly classified 83% of patients and 87% of controls, corresponding to a total accuracy of 85% (p-value = 0.01, permutation test with 100 repetitions, Table 1). In comparison, correlation, inverse covariance and partial correlation-based measures again did not perform better than chance. Correlation-based features correctly classified 63% of patients and 50% of controls, total accuracy of 57% (p-value > 0.05, permutation test with 100 repetitions). Partial correlation correctly classified 53% of patients and 43% of controls, corresponding to an accuracy of 48% (p-value > 0.05, permutation test with 100 repetitions). Full inverse covariance-based features correctly classified only 47% of patients and 53% of controls, total accuracy of 50% (p-value > 0.05, permutation test with 100 repetitions). These results are summarized in Table 1.

The results obtained with the L2-norm SVM are summarized in Table 2. For the block-related dataset, L2-norm SVM using sparse inverse covariances as features yielded an accuracy of 78% (with 80% sensitivity and 77% specificity; p-value = 0.01, permutation test with 100 repetitions, Table 2). However, for this dataset, none of the other features yielded significant predictive accuracies. Full correlation yielded an accuracy of 60% (p-value > 0.05, permutation test with 100 repetitions); partial correlation yielded an accuracy of 58%, while full inverse covariance-based features yielded an accuracy of only 40% (p-value > 0.05, permutation test with 100 repetitions).

#### Pattern stability/reproducibility

Similarly to the results obtained for the event-related dataset, for the block-related data L1-norm SVMs again provided more stable patterns than L2-norm SVMs (57.25% ± 3.45% compared to 25.81% ± 0.45%, respectively) using the sparse inverse covariance as features. The same seems to hold for all other connectivity measures.

#### Pattern interpretation

**Fig. 5:**
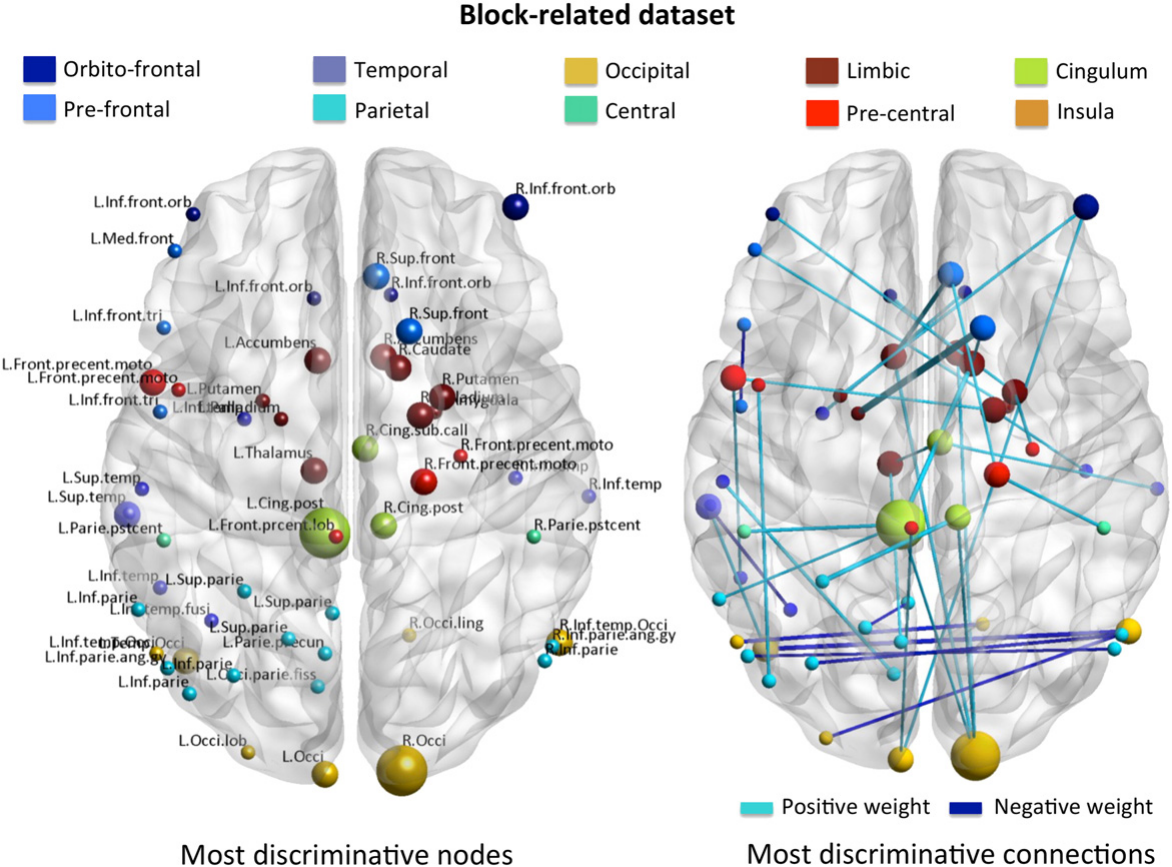
A. Set of most discriminative nodes for the block-related fMRI dataset. The size of the node is proportional to the number of connections that link the corresponding node to others (visualized with BrainNet Viewer); B. Set of most discriminative connections (weight vector) for the block-related dataset. The width of the connection is proportional to the absolute value of the corresponding weight.

**Fig. 6:**
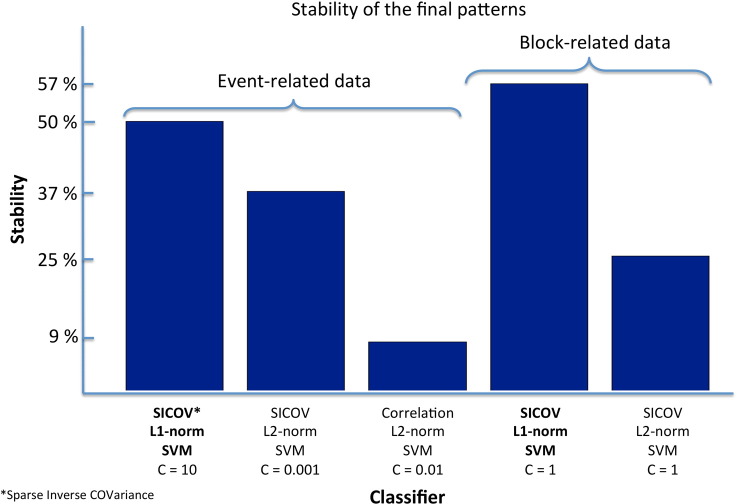
Stability of the final patterns (obtained by retraining the classification models, with significant accuracy, using the entire dataset as described in the Methods section).

The distributed connectivity response that discriminated between patients with MDD symptoms and healthy participants in the block-related fMRI dataset comprised a set of 45 (out of 9316 possible) connections (Table 4 and Fig. 5B), after re-training the SVM using all data and a *C* parameter of 1 (obtained as described in the Methods section). The λ parameter (Eq. (1)) did not vary across folds and we therefore used the optimal value of 0.01 to re-train the model. To test the significance of these connections we ran a permutation test on the weights (100 samples) as described in Methods. The stability of the final patterns (output of Eq. (6)) obtained with L1 and L2-norm SVM, measured using a leave-one-subject-per-group-out CV and C = 1 for both models, is plotted in Fig. 6.

The resulting network with 38 statistically significant connections (p-value < 0.05 FDR corrected) is shown in Table 4 and Fig. 5B. Again this network highlights *limbic-cortical*, in particular *striatal-cortical*, circuitry and include links between the: left pallidum and right superior frontal cortex; left nucleus accumbens and right superior frontal cortex; left nucleus accumbens and right occipital cortex; right putamen and right precentral (motor) cortex; right nucleus accumbens and right occipital cortex; right caudate and right superior frontal cortex; right palligum and left precentral (motor) cortex; left thalamus and right subcallosal cortex; right amygdala and left medial frontal cortex; right pallidum and right inferior temporal cortex; left thalamus and left precuneus. *Limbic-orbitofrontal connections* include: right nucleus accumbens and left orbito-frontal cortex; left putamen and right orbito-frontal cortex; right putamen and left orbito-frontal cortex. In addition, the network also highlights *cingulate-cortical connections* (right posterior cingulate cortex and left superior parietal cortex; left posterior cingulate cortex and left postcentral cortex; right posterior cingulate cortex and right occipital cortex; left posterior cingulate cortex and left inferior parietal cortex; left posterior cingulate cortex and right occipital cortex), as well as *limbic-cingulate connections* (right caudate and left posterior cingulate cortex).

The most discriminative nodes (with 4 connections each) for the block-related fMRI dataset were located in the left posterior cingulate cortex and right occipital cortex. The rest of the nodes connected to two or only one node each.

It is again important to emphasize here that all 45 connections and corresponding regions contributed to the predictions. The full list of connections and coordinates in the atlas can be found in Table 4.

## Discussion

In this paper, we presented a novel connectivity-based discriminative framework combining sparse inverse covariance-based features and L1-norm regularized linear SVMs. In addition, our framework was optimized using not only the predictive accuracy, as is the common practice, but also the reproducibility/stability of the models. We applied this technique to two (one event-related and one block-related) fMRI datasets acquired with an emotional facial processing task from two different samples of patients with symptoms of MDD and matched healthy participants.

Our modeling framework provided similar or more powerful predictions than whole-brain (voxel-based) non-sparse classification models applied to the same data (Fu et al., 2008; Hahn et al., 2011), with the advantage of being more straightforwardly interpretable in terms of the underlying neurobiology of MDD. In particular, accuracy reported by Fu et al. (2008), using all facial stimuli, reached 77%, compared to 79% obtained with our approach. Similarly, the highest accuracy obtained by Hahn et al. (2011) using facial expression stimuli did not reach 70%, while we obtained 85% accuracy using the same data. The fact that we obtained lower sensitivity and specificity for the first dataset could be due to the smaller sample size of these data (only 19 subjects in each group, compared to 30 in the second dataset) and also the fact that we had longer time-series for the second dataset (256 time-points compared to 180).

To test whether other connectivity-based features yielded similar results we compared the inverse covariance to commonly used correlation and partial correlation based metrics. However, we obtained not only better classification results using the sparse inverse covariance matrices but also these were the only models for which the predictions were significantly different from chance. None of the compared features (correlation, partial correlation and full inverse covariance) yielded significant classification results. For this reason we were unable to compare the patterns obtained with the sparse inverse covariance to the patterns obtained with the other approaches. In addition, we note here that although the mean non-sparse covariance matrices shown in Fig. 2 suggest that there are differences between the groups, we did not find any significant results supporting this observation, both using univariate statistics (two-sample *t*-test on all features corrected for multiple comparisons using False Discovery Rate, results not shown) and multivariate pattern recognition models (Table 1). We also tested if the total number of non-zero connections in the sparse inverse covariance matrices differed between groups. Again, we did not find any significant results for both datasets (p-value = 0.10 for the event-related data and p-value = 0.37 for the block-related data). For these reasons and given our a priori hypothesis, we conclude that it is the multivariate pattern of non-zero and zero connections comprising the sparse inverse covariance matrices (estimated with graphical lasso) that allows us to discriminate between patients and controls.

To further substantiate our initial hypothesis that task induced neuronal processing involves only a discrete number of connections between brain regions, from which only a subset is affected by depression we compared our results with the ones obtained with L2-norm SVMs (a non-sparse classifier). The non-sparse SVMs did not provide better accuracy, nor higher stability/reproducibility, than the sparse classifiers (Table 2 and Fig. 6) therefore supporting our initial hypothesis.

The most discriminative features revealed by our framework are consistent with the recent literature on functional connectivity in depression (Anand et al., 2005; Greicius et al., 2007; Furman et al., 2011; Wu et al., 2011; Fang et al., 2012; Ajilore et al., 2014), and highlight differences between the groups in cortico-limbic (in particular cortico-striatal) and cortico-cingulate circuitry associated with emotional regulation. A recent review on univariate and connectivity results from studies using fMRI data from MDD patients and emotional facial processing tasks has found a set of consistent regions that are though to be responsible for both a negative and positive bias in patients when processing these tasks (Stuhrmann et al., 2011). These regions comprise limbic areas, such as the amygdala, hippocampus, insula, thalamus, and the striatum. Abnormal activity in MDD patients is also reported in cingulate cortex, motor, pre/orbito-frontal and temporal regions (Stuhrmann et al., 2011). In addition, evidence is accumulating regarding the importance of striatal-cortical and cingulate-cortical connections in populations with depression-related symptoms (Ring and Serra-Mestres, 2002; Furman et al., 2011; Gabbay et al., 2013). The most discriminative connections yielded by our approach therefore overlap considerably with these findings for both datasets.

Both datasets used in this work contain patients with depressive symptoms and an emotion-processing task. However they differ in a number of aspects: i) the first dataset has an event-related design, while the second has a block-related one. These two types of design have been shown to engage different networks in emotion related paradigms (Schäfer et al., 2005; Bühler et al., 2008); ii) the first dataset contains a set of patients with a fairly homogeneous diagnosis of major depressive disorder (score of at least 18 on Hamilton Rating Scale for Depression), while the patients from the second dataset presented varying degrees of depressive symptoms (from severe to almost symptom free) and were diagnosed with recurrent depressive disorder, depressive episodes, or bipolar affective disorder; and iii) patients from the first dataset were not medicated at the time of the experiment, while patients from the second dataset were recruited on a variety of medications. For these reasons we expected slightly different discriminative patterns. Nevertheless, we expected these patterns to highlight differences in cortico-limbic/cingulate circuitry associated with emotional regulation for both datasets, as was indeed observed.

One limitation of our approach, and network-based approaches in general, is that it depends highly on the anatomical atlas used to segment the regions. Some atlases might be better than others for a given problem, depending on how well the anatomical regions overlap with functional regions determined by the data. Here we relied on an inter-subject atlas of sulci probabilities (Perrot et al., 2009), which has been shown to provide good anatomical regions for fMRI analyses (Keller et al., 2009). However, since this issue affects all atlas-based connectivity approaches, future connectivity models (both univariate and multivariate) would benefit from a thorough investigation into this issue. This investigation is however, outside of the scope of this work. An alternative to the atlas approach is to apply a clustering algorithm on the data to define brain regions based on their functional similarity, for example see Bellec et al. (2010) and Craddock et al. (2012). However, these approaches can be computationally expensive and the choice of number of clusters can be somewhat arbitrary.

Even though sparse models in general aim to facilitate interpretation, in practice they can be highly irreproducible under certain conditions, as shown in Rasmussen et al. (2012), which can hinder their main goal. Here we introduced the stability (pattern overlap) measure proposed by Baldassarre et al. (2012) as an additional criterion to optimize our learning framework. This way the sparsity parameter of the predictive model (the L1-norm SVM) was based not only on the accuracy of the model but also on how stable the patterns were across cross-validation folds. This joint optimization procedure was proposed by Rasmussen et al. (2012). Here we chose to assess reproducibility across cross-validation folds, instead of using the proposed half-split subsampling approach (Rasmussen et al., 2012), due to the relatively small sample size of our datasets. Even though this method allowed us to more confidently interpret the resulting sparse patterns, further investigation into the relation between different measures of sparsity and accuracy, would benefit the use of sparse predictive models in general (Ryali et al., 2012).

In addition, the L1-norm penalty used in both the inverse covariance and the linear SVMs does not take into account underlying structure in the features. In other words, in the presence of two correlated features the L1-norm will select only one of these features. To mitigate this issue, one can replace the L1-norm by a more general penalty function. Examples include the Elastic-Net penalty (Ryali et al., 2012), which is a linear combination of L1 and L2-norms, and a group-LASSO penalty function (Friedman et al., 2010), which selects not only features individually but also groups of correlated features.

Here we applied our modeling approach to task-based fMRI from MDD patients and healthy participants. However, our approach is entirely general and can be easily applied to other types of fMRI data (task-based as well as resting state), other data modalities (e.g. Arterial Spin Labeling) and other classification problems (both clinic and neuroscience oriented).

To conclude, we showed that it is possible to discriminate patients with major depression from healthy controls, using sparse network-based predictive models and fMRI data acquired during a task involving emotional facial processing. When compared to whole-brain voxel-based analyses on the same data (using all emotional stimuli and a non-sparse classifier) (Fu et al., 2008; Hahn et al., 2011) and correlation-based metrics, our approach provided higher accuracy, while revealing a stable distributed network of cortical and striatal/cingulate regions underlying discriminative differences in brain connectivity between MDD and healthy participants.

## Figures and Tables

**Table 1:** Classification accuracies, sensitivity and specificity, sparsity and stability for all the network-based models compared, obtained with L1-norm SVM. The ⁎ denotes a p-value < 0.05. p-values were obtained using permutation tests, as described in the main text.

Classification results L1-norm SVM						
Features	Accuracy (%)	Accuracy p-value	Sensitivity (%)	Specificity (%)	Sparsity (%)	Stability (%)
*Event-related fMRI dataset*						
Sparse inverse covariance	**78.95**	**0.02**⁎	**68.42**	**89.47**	**0.47 ± 0.16**	**54.02 ± 6.00**
Full inverse covariance	28.95	> 0.05	31.58	26.32	6.60 ± 4.85	78.01 ± 7.64
Correlation	65.79	= 0.05	84.21	47.37	1.37 ± 1.25	61.73 ± 3.39
Partial correlation	34.21	> 0.05	47.37	21.05	3.50 ± 1.79	75.50 ± 4.02

*Block-related fMRI dataset*						
Sparse inverse covariance	**85.00**	**0.01**⁎	**83.33**	**86.67**	**0.60 ± 0.60**	**57.25 ± 3.45**
Full inverse covariance	50.00	> 0.05	46.67	53.33	0.83 ± 0.56	60.62 ± 4.66
Correlation	56.67	> 0.05	63.33	50.00	1.50 ± 1.57	51.53 ± 4.77
Partial correlation	48.33	> 0.05	53.33	43.33	2.03 ± 1.34	65.95 ± 5.14

⁎ p-Value < 0.05.

**Table 2:** Classification accuracies, sensitivity and specificity, sparsity and stability for all the network-based models compared, obtained with L2-norm SVM. The ⁎ denotes a p-value < 0.05. p-values were obtained using permutation tests, as described in the main text.

Classification results L2-norm SVM						
Features	Accuracy (%)	Accuracy p-value	Sensitivity (%)	Specificity (%)	Sparsity (%)	Stability (%)
*Event-related fMRI dataset*						
Sparse inverse covariance	**73.68**	**0.01**⁎	**73.68**	**73.68**	**–**	**37.81 ± 0.26**
Full inverse covariance	44.73	> 0.05	47.37	42.11	–	9.90 ± 0.78
Correlation	**68.42**	**0.02**⁎	**84.21**	**52.63**	**–**	**9.29 ± 1.56**
Partial correlation	65.78	> 0.05	73.68	57.89	–	11.33 ± 0.16

*Block-related fMRI dataset*						
Sparse inverse covariance	**78.33**	**0.01**⁎	**80.00**	**76.67**	**–**	**25.81 ± 0.45**
Full inverse covariance	40.00	> 0.05	20.00	60.00	–	12.81 ± 0.89
Correlation	60.00	> 0.05	86.67	33.33	–	8.61 ± 2.17
Partial correlation	58.33	> 0.05	53.33	63.33	–	10.93 ± 0.78

⁎ p-value < 0.05.

**Table 3:** The set of most discriminative connections for the event-related fMRI dataset. These connections correspond to the (59 out of 9316) non-zero entries of the weight vector output by the linear L1-norm SVM that survived permutation testing and FDR correction (p-value < 0.05, 100 samples). The coordinates shown correspond to the atlas coordinates. The atlas regions have been relabeled for easier interpretation. The full list of regions, as well as the original and new labels can be found in the Supplementary material.

Event-related fMRI dataset: most discriminative connections	
Region i [x y z] mm	Region j [x y z] mm
R.occi.ling [16, − 64, − 6]	R.occi.lob [35, − 92, 0]
L.putamen [− 24, 0, 0]	R.front.prcent.lob [5, − 37, 62]
R.sup.parie [30, − 50, 66]	L.front.prcent.lob [− 4, − 37, 64]
R.mid.front [37, 43, 31]	L.cing.ant [− 6, 22, 33]
R.inf.front.tri [48, 27, 2]	R.inf.front.orb [36, 16, − 15]
R.front.precent.motor [30, − 15, 67]	L.inf.front.orb [− 43, 51, 8]
L.inf.parie [− 62, − 43, 39]	L.parie.syl [− 63, − 3, 21]
R.sup.parie [6, − 54, 34]	L.cing.sub.call [− 2, − 21, 26]
R.sup.front [18, 62, 27]	R.sup.front [16, 19, 64]
R.insula [44, 1, 5]	R.inf.front.tri [48, 27, 2]
R.palladium [19, − 4, − 1]	R.inf.front.tri [53, 19, 10]
L.sup.front.prcent.lob.SMA [− 6, − 24, 66]	L.cing.ant [− 6, 22, 33]
L.inf.front.orb [− 4, 47, − 3]	L.inf.front.orb [− 29, 39, − 8]
R.thalamus [11, − 18, 6]	R.cing.ant [7, 25, 31]
R.hippocampus [26, − 21, − 13]	L.inf.parie [− 32, − 75, 39]
R.inf.temp [65, − 26, − 20]	R.inf.temp [55, − 44, − 22]
L.putamen [− 24, 0, 0]	R.sup.front [29, 21, 55]
R.inf.parie.ang.gy [55, − 67, 18]	L.occi.cun [− 4, − 95, 14]
L.inf.temp [− 44, -24, − 28]	L.inf.front.orb [− 36, 14, − 18]
L.insula [− 41, 1, 4]	L.parie.pstcent [− 51, − 38, 48]
R.amygdala [23, − 3, − 18]	R.inf.temp.fusi [38, − 61, − 20]
L.inf.front.orb [− 29, 39, − 8]	R.mid.front [25, 63, 4]
L.caudate [− 12, 8, 10]	L.sup.front [− 16, 63, 26]
R.sup.parie [6, − 54, 34]	R.cing.sub.call [4, − 13, 27]
L.front.precent.moto [− 41, − 10, 57]	L.parie.pstcent [− 42, − 27, 54]
L.occi.parie.fiss [− 9, − 78, 22]	L.occi.temp.fusi [− 26, − 53, − 16]
R.med.front [50, 42, 12]	R.insula [44, 1, 5]
R.sup.front [29, 21, 55]	R.mid.front [37, 43, 31]
R.palladium [19, − 4, − 1]	R.sup.front [16, 19, 64]
R.front.precent.moto [49, 7, 45]	R.sup.temp [59, − 20, 16]
R.putamen [25, 1, 0]	R.inf.temp [55, − 44, − 22]
R.caudate [13, 9, 10]	L.sup.front [− 16, 63, 26]
R.thalamus [11, − 18, 6]	L.front.prcent.lob [− 4, − 37, 64]
L.amygdala [− 23, − 4, − 18]	L.front.precent.moto [− 30, − 17, 66]
R.sup.temp [63, − 25, 0]	L.sup.temp [− 57, − 24, 13]
L.inf.parie.spmarg [− 64, − 25, 28]	L.sup.temp [− 57, − 24, 13]
R.inf.temp [45, − 21, − 29]	R.mid.front [25, 63, 4]
L.inf.parie.ang.gy [− 50, − 73, 15]	R.cing.sub.call [4, − 13, 27]
L.sup.front [− 16, 19, 64]	R.cing.ant [7, 25, 31]
L.inf.front [− 48, 26, 28]	L.insula [− 41, 1, 4]
R.sup.parie [6, − 54, 34]	L.occi.ling [− 10, − 76, 0]
L.sup.temp [− 61, − 31, 0]	L.parie.pstcent [− 42, − 27, 54]
L.sup.temp [− 61, − 31, 0]	R.insula [44, 1, 5]
L.putamen [− 24, 0, 0]	L.sup.front [− 27, 19, 54]
R.insula [44, 1, 5]	R.cing.ant [7, 25, 31]
R.cing.sub.call [4, − 13, 27]	R.occi.ling [13, − 72, 3]
R.inf.temp [55, − 44, − 22]	L.sup.temp [− 57, − 24, 13]
L.inf.front.tri [− 52, − 3, 11]	R.inf.front.tri [53, 19, 10]
L.sup.front.prcent.lob.sma [− 6, − 24, 66]	R.front.precent.moto [30, − 15, 67]
L.palladium [− 19, − 5, − 1]	L.cing.ant [− 6, 22, 33]
L.inf.temp [− 52, − 51, − 22]	L.mid.front [− 23, 61, 6]
L.amygdala [− 23, − 4, − 18]	R.sup.front [29, 21, 55]
L.front.precent.moto [− 54, 5, 29]	L.inf.front [− 48, 26, 28]
R.inf.temp.occi [57, − 66, − 1]	L.occi.parie.fiss [− 9, − 78, 22]
L.accumbens [− 9, 11, − 6]	L.front.precent.moto [− 30, − 17, 66]
R.inf.temp [65, − 26, − 20]	R.sup.temp [59, − 20, 16]

**Table 4:** The set of most discriminative connections for the block-related fMRI dataset. These connections correspond to the (38 out of 9316) non-zero entries of the weight vector output by the linear L1-norm SVM that survived permutation testing and FDR correction (p-value < 0.05, 100 samples). The coordinates shown correspond to the atlas coordinates. The atlas regions have been relabeled for easier interpretation. The full list of regions, as well as the original and new labels can be found in the Supplementary material.

Block-related fMRI dataset: most discriminative connections	
Region i [x y z] mm	Region j [x y z] mm
L. palladium [− 19, − 5, − 1]	R. sup. front [16, 19, 64]
R. caudate [13, 9, 10]	L. cing. post [− 7, − 36, 51]
L. sup. parie [− 29, − 52, 65]	R. cing. post [9, − 34, 51]
R. inf. temp. occi [57, − 66, − 1]	L. temp. occi [− 45, − 71, − 17]
L. accumbens [− 9, 11, − 6]	R. sup. front [7, 34, 30]
L. inf. temp. occi [− 53, − 69, − 3]	R. occi. ling [16, − 64, − 6]
L. occi. parie. fiss [− 9, − 78, 22]	L. sup. temp [− 57, − 24, 13]
L. parie. pstcent [− 51, − 38, 48]	L. cing. post [− 7, − 36, 51]
R. inf. parie. ang. gy [55, − 67, 18]	L. inf. parie [− 32,− 75, 39]
L. sup. temp [− 61, − 31, 0]	L. inf. temp. fusi [− 38, − 60, − 23]
L. accumbens [− 9, 11, − 6]	R. occi [14, − 101, − 8]
R. front. precent. motor [20, − 22, 73]	R. parie. pstcent [50, − 37, 52]
R. inf. temp. occi [57, − 66, − 1]	L. occi. lob [− 28, − 96, − 3]
L. front. precent. motor [− 47, 3, 44]	L. inf. parie [− 44, − 80, 33]
R. putamen [25, 1, 0]	R. front. precent. motor [30, − 15, 67]
R. accumbens [9, 12, − 6]	L. inf. front. orb [− 10, 28, − 16]
L. inf. temp [− 29, − 5, − 35]	R. inf. front. orb [11, 29, − 15]
L. putamen [− 24, 0, 0]	R. inf. front. orb [45, 53, 7]
L. sup. temp [− 61, − 31, 0]	L. temp. occi [− 45, − 71, − 17]
R. occi [14, − 101, − 8]	R. cing. post [9, − 34, 51]
R. accumbens [9, 12, − 6]	R. occi [14, − 101, − 8]
L. inf. parie. ang. gy [− 50, − 73, 15]	R. inf. parie [53, − 71, 35]
L. inf. front. tri [− 52, − 3, 11]	L. inf. front. tri [− 51, 20, 11]
L. sup. parie [− 5, − 58, 31]	L. sup. parie [− 17, − 65, 63]
R. caudate [13, 9, 10]	R. sup. front [16, 19, 64]
L. front. precent. moto [− 54, 5, 29]	L. inf. temp [− 52, − 51, − 22]
R. inf. temp [65, –26, − 20]	R. cing. sub. call [4, − 13, 27]
R. palladium [19, − 4, − 1]	L. front. precent. moto [− 54, 5, 29]
L. thalamus [− 10, − 19, 6]	R. cing. sub. call [4, − 13, 27]
L. occi [− 7, − 102, − 12]	R. inf. front. orb [45, 53, 7]
L. occi [− 7, − 102, − 12]	L. front. prcent. lob [− 4, − 37, 64]
R. putamen [25, 1, 0]	L. inf. front. orb [− 43, 51, 8]
R. amygdala [23, − 3, − 18]	L. med. front [− 48, 41, 11]
L. inf. parie [− 58, − 57, 39]	L. cing. post [− 7, − 36, 51]
R. palladium [19, − 4, − 1]	R. inf. temp [45, − 21, − 29]
R. front. precent. moto [20, − 22, 73]	R. sup. front [7, 34, 30]
L. thalamus [− 10, − 19, 6]	L. parie. precun [− 7, − 69, 47]
R. occi [14, − 101, − 8]	L. cing. post [− 7, − 36, 51]

## Appendix A Supplementary data

Table S1: Atlas coordinates, original atlas label and new label for each region.

## Footnotes

1 BrainVISA atlas: http://lnao.lixium.fr/spip.php?article=229.

2 Harvard-Oxford atlas: http://fsl.fmrib.ox.ac.uk/fsl/fslwiki/Atlases.

3 We used the R software package *glasso*: http://cran.r-project.org/web/packages/glasso/.

4 LIBLINEAR software package: http://www.csie.ntu.edu.tw/~cjlin/liblinear/.

5 LIBSVM software package: http:www.csie.ntu.edu.tw/~cjlin/libsvm/.

6 We only plot the discriminative connections obtained with L1-norm SVM for the sparse inverse covariance model because this was the only model with significant predictive accuracy (p-value < 0.05). Models for which we could not find a significant predictive relationship between brain connectivity and the subjects' labels were not further interpreted.
