# Supplementary material for: Sparse network-based models for patient classification using fMRI
Source: Neuroimage. 2015 Jan 15;105:493–506. doi: 10.1016/j.neuroimage.2014.11.021 (PMC4275574; doi:10.1016/j.neuroimage.2014.11.021)
Supplement: Supplementary file 1 — Table S1: Atlas coordinates, original atlas label and new label for each region. [file mmc1.docx]

**Supplementary material**

**x y z Original atlas label** **New label**

-36 14 -18 Left anterior lateral fissure L.Inf.front.orb

36 16 -15 Right anterior lateral fissure R.Inf.front.orb

-57 -24 13 Left posterior lateral fissure L.Sup.temp

59 -20 16 Right posterior lateral fissure R.Sup.temp

-46 26 3 Left anterior ramus of the lateral fissure L.Inf.front.tri

48 27 2 Right anterior ramus of the lateral fissure R.Inf.front.tri

-51 20 11 Left ascending ramus of the lateral fissure L.Inf.front.tri

53 19 10 Right ascending ramus of the lateral fissure R.Inf.front.tri

-55 13 18 Left diagonal ramus of the lateral fissure L.Inf.front.oper

58 15 18 Right diagonal ramus of the lateral fissure R.Inf.front.oper

-64 -25 28 Left retro central transverse ramus of the lateral fissure L.Inf.parie.spmarg

65 -23 33 Right retro central transverse ramus of the lateral fissure R.Inf.parie.spmarg

-52 -3 11 Left anterior sub-central ramus of the lateral fissure L.Inf.front.tri

52 -4 13 Right anterior sub-central ramus of the lateral fissure R.Inf.front.tri

-54 -18 17 Left posterior sub-central ramus of the lateral fissure L.Parie.pstcent

55 -17 19 Right posterior sub-central ramus of the lateral fissure R.Parie.pstcent

-6 22 33 Left calloso-marginal anterior fissure L.Cing.ant

7 25 31 Right calloso-marginal anterior fissure R.Cing.ant

-7 -36 51 Left calloso-marginal posterior fissure L.Cing.post

9 -34 51 Right calloso-marginal posterior fissure R.Cing.post

-10 -76 0 Left calcarine fissure L.Occi.ling

13 -72 3 Right calcarine fissure R.Occi.ling

-26 -53 -16 Left collateral fissure L.Occi.temp.fusi

28 -52 -14 Right collateral fissure R.Occi.temp.fusi

-51 -38 48 Left superior postcentral intraparietal superior sulcus L.Parie.pstcent

50 -37 52 Right superior postcentral intraparietal superior sulcus R.Parie.pstcent

-32 -75 39 Left intraparietal sulcus L.Inf.parie

36 -72 42 Right intraparietal sulcus R.Inf.parie

-58 -57 39 Left primary intermediate ramus of the intraparietal sulcus L.Inf.parie

64 -47 35 Right primary intermediate ramus of the intraparietal sulcus R.Inf.parie

-44 -80 33 Left secondary intermediate ramus of the intraparietal sulcus L.Inf.parie

53 -71 35 Right secondary intermediate ramus of the intraparietal sulcus R.Inf.parie

-9 -78 22 Left parieto-occipital fissure L.Occi.parie.fiss

13 -75 23 Right parieto-occipital fissure R.Occi.parie.fiss

-41 1 4 Left insula L.Insula

44 1 5 Right insula R.Insula

-28 -96 -3 Left lobe occipital L.Occi.lob

35 -92 0 Right lobe occipital R.Occi.lob

-4 -37 64 Left paracentral lobule central sulcus L.Front.prcent.lob

5 -37 62 Right paracentral lobule central sulcus R.Front.prcent.lob

-42 -27 54 Left central sulcus L.Parie.pstcent

43 -25 56 Right central sulcus R.Parie.pstcent

-63 -3 21 Left central sylvian sulcus L.Parie.Syl

65 -2 21 Right central sylvian sulcus R.Parie.Syl

-2 -21 26 Left subcallosal sulcus L.Cing.sub.call

4 -13 27 Right subcallosal sulcus R.Cing.sub.call

-4 -95 14 Left cuneal sulcus L.Occi.cun

10 -95 15 Right cuneal sulcus R.Occi.cun

-48 26 28 Left inferior frontal sulcus L.Inf.front

50 26 29 Right inferior frontal sulcus R.Inf.front

-48 41 11 Left anterior inferior frontal sulcus L.Med.front

50 42 12 Right anterior inferior frontal sulcus R.Med.front

-5 35 28 Left internal frontal sulcus L.Sup.front

7 34 30 Right internal frontal sulcus R.Sup.front

-35 40 33 Left intermediate frontal sulcus L.Mid.front

37 43 31 Right intermediate frontal sulcus R.Mid.front

-23 61 6 Left marginal frontal sulcus L.Mid.front

25 63 4 Right marginal frontal sulcus R.Mid.front

-16 19 64 Left median frontal sulcus L.Sup.front

16 19 64 Right median frontal sulcus R.Sup.front

-43 51 8 Left orbital frontal sulcus L.Inf.front.orb

45 53 7 Right orbital frontal sulcus R.Inf.front.orb

-16 63 26 Left polar frontal sulcus L.Sup.front

18 62 27 Right polar frontal sulcus R.Sup.front

-27 19 54 Left superior frontal sulcus L.Sup.front

29 21 55 Right superior frontal sulcus R.Sup.front

-62 -43 39 Left sulcus of the supra-marginal gyrus L.Inf.parie

-14 -65 -8 Left anterior intralingual sulcus L.Occi.ling

16 -64 -6 Right anterior intralingual sulcus R.Occi.ling

-8 -82 -13 Left posterior intra-lingual sulcus L.Occi.ling

11 -80 -10 Right posterior intra-lingual sulcus R.Occi.ling

-44 -24 -28 Left anterior occipito-temporal lateral sulcus L.Inf.temp

45 -21 -29 Right anterior occipito-temporal lateral sulcus R.Inf.temp

-38 -60 -23 Left internal occipito-temporal lateral sulcus L.Inf.temp.fusi

38 -61 -20 Right internal occipito-temporal lateral sulcus R.Inf.temp.fusi

-52 -51 -22 Left median occipito-temporal lateral sulcus L.Inf.temp

55 -44 -22 Right median occipito-temporal lateral sulcus R.Inf.temp

-45 -71 -17 Left posterior occipito-temporal lateral sulcus L.Temp.Occi

47 -67 -17 Right posterior occipito-temporal lateral sulcus R.Temp.Occi

-7 -102 -12 Left occipito-polar sulcus L.Occi

14 -101 -8 Right occipito-polar sulcus R.Occi

-10 28 -16 Left olfactory sulcus L.Inf.front.orb

11 29 -15 Right olfactory sulcus R.Inf.front.orb

-29 39 -8 Left orbital sulcus L.Inf.front.orb

32 38 -7 orbital sulcus R.Inf.front.orb

-7 -69 47 Left internal parietal sulcus L.Parie.precun

8 -65 48 Right internal parietal sulcus R.Parie.precun

-17 -65 63 Left superior parietal sulcus L.Sup.parie

20 -66 63 Right superior parietal sulcus R.Sup.parie

-10 -78 35 Left transverse parietal sulcus L.Sup.parie

15 -80 38 Right transverse parietal sulcus R.Sup.parie

-54 5 29 Left inferior precentral sulcus L.Front.precent.motor

56 7 29 Right inferior precentral sulcus R.Front.precent.motor

-47 3 44 Left intermediate precentral sulcus L.Front.precent.motor

49 7 45 Right intermediate precentral sulcus R.Front.precent.motor

-30 -17 66 Left marginal precentral sulcus L.Front.precent.motor

30 -15 67 Right marginal precentral sulcus R.Front.precent.motor

-20 -24 72 Left median precentral sulcus L.Front.precent.motor

20 -22 73 Right median precentral sulcus R.Front.precent.motor

-41 -10 57 Left superior precentral sulcus L.Front.precent.motor

43 -7 57 Right superior precentral sulcus R.Front.precent.motor

-29 -52 65 Left superior postcentral sulcus L.Sup.parie

30 -50 66 Right superior postcentral sulcus R.Sup.parie

-4 47 -3 Left inferior rostral sulcus L.Inf.front.orb

5 46 -5 Right inferior rostral sulcus R.Inf.front.orb

-29 -5 -35 Left rhinal sulcus L.Inf.temp

30 -4 -34 Right rhinal sulcus R.Inf.temp

-62 -27 -21 Left anterior inferior temporal sulcus L.Inf.temp

65 -26 -20 Right anterior inferior temporal sulcus R.Inf.temp

-53 -69 -3 Left posterior inferior temporal sulcus L.Inf.temp.Occi

57 -66 -1 Right posterior inferior temporal sulcus R.Inf.temp.Occi

-45 9 -32 Left polar temporal sulcus L.Temp.pole

45 10 -32 Right polar temporal sulcus R.Temp.pole

-61 -31 0 Left superior temporal sulcus L.Sup.temp

63 -25 0 Right superior temporal sulcus R.Sup.temp

-54 -65 26 Left anterior terminal ascending branch L.Inf.parie.ang.gy

of the superior temporal sulcus

59 -54 28 Right anterior terminal ascending branch R.Inf.parie.ang.gy

of the superior temporal sulcus

-50 -73 15 Left posterior terminal ascending branch L.Inf.parie.ang.gy

of the superior temporal sulcus

55 -67 18 Right posterior terminal ascending branch R.Inf.parie.ang.gy

of the superior temporal sulcus

-6 -24 66 Left paracentral sulcus L.Sup.front.prcent.lob.SMA

7 -29 65 Right paracentral sulcus R.Sup.front.prcent.lob.SMA

-5 -58 31 Left sub-parietal sulcus L.Sup.parie

6 -54 34 Right sub-parietal sulcus R.Sup.parie

-10 -19 6 Left Thalamus L.Thalamus

-12 8 10 Left Caudate L.Caudate

-24 0 0 Left Putamen L.Putamen

-19 -5 -1 Left Pallidum L.Palladium

-24 -22 -14 Left Hippocampus L.Hippocampus

-23 -4 -18 Left Amygdala L.Amygdala

-9 11 -6 Left Accumbens L.Accumbens

11 -18 6 Right Thalamus R.Thalamus

13 9 10 Right Caudate R.Caudate

25 1 0 Right Putamen R.Putamen

19 -4 -1 Right Pallidum R.Palladium

26 -21 -13 Right Hippocampus R.Hippocampus

23 -3 -18 Right Amygdala R.Amygdala

9 12 -6 Right Accumbens R.Accumbens
